# Wastewater Surveillance for SARS-CoV-2 in Northern Italy: An Evaluation of Three Different Gene Targets

**DOI:** 10.3390/microorganisms13020236

**Published:** 2025-01-22

**Authors:** Giulio Mannarà, Marianna Martinelli, Chiara Giubbi, Michelle Rizza, Eleonora Giordano, Federica Perdoni, Erika Bruno, Annalisa Morella, Arianna Azzellino, Andrea Turolla, Ramon Pedrini, Francesca Malpei, Giuseppina La Rosa, Elisabetta Suffredini, Danilo Cereda, Emanuela Ammoni, Simone Villa, Francesca Pregnolato, Marialuisa Lavitrano, Andrea Franzetti, Rosario Musumeci, Clementina E. Cocuzza

**Affiliations:** 1 School of Medicine and Surgery, University of Milano-Bicocca, 20900 Monza, Italy; giulio.mannara@unimib.it (G.M.); marianna.martinelli@unimib.it (M.M.); chiara.giubbi@unimib.it (C.G.); m.rizza8@campus.unimib.it (M.R.); eleonora.giordano@unimib.it (E.G.); federica.perdoni@unimib.it (F.P.); marialuisa.lavitrano@unimib.it (M.L.); 2 Department of Earth and Environmental, Sciences—DISAT, University of Milano-Bicocca, 20126 Milan, Italy; erika.bruno@unimib.it (E.B.); andrea.franzetti@unimib.it (A.F.); 3 Società per l’Ecologia e l’Ambiente (SECAM) S.P.A., 23100 Sondrio, Italy; annalisa.morella@secam.net; 4 Department of Civil and Environmental Engineering, Politecnico di Milano, 20133 Milan, Italy; arianna.azzellino@polimi.it (A.A.); andrea.turolla@polimi.it (A.T.); ramon.pedrini@polimi.it (R.P.); francesca.malpei@polimi.it (F.M.); 5 National Center for Water Safety (CeNSia), Istituto Superiore di Sanità, 00161 Rome, Italy; giuseppina.larosa@iss.it; 6 Department of Food Safety, Nutrition and Veterinary Public Health, Istituto Superiore di Sanità, 00161 Rome, Italy; elisabetta.suffredini@iss.it; 7 UO Prevenzione, DG Welfare, Regione Lombardia, 20124 Milan, Italy; danilo_cereda@regione.lombardia.it (D.C.); emanuela_ammoni@regione.lombardia.it (E.A.); francesca_pregnolato@regione.lombardia.it (F.P.); 8 Department of Computer Science, University of Milan, 20133 Milan, Italy; simone.villa@unimi.it

**Keywords:** wastewater surveillance, WBE, SARS-CoV-2, ORF1b, N1, N3, quantification, frequency, Italy, temporal dynamics

## Abstract

Wastewater-based epidemiology has emerged as a complementary tool for the monitoring of COVID-19 pandemic waves and for the circulation of viral variants. The selection, standardization, and dynamics of different SARS-CoV-2 RNA targets in wastewater requires further investigation. In the present study, 106 wastewater samples were collected over a 24-month period from the wastewater treatment plant of Sondrio, north Italy, and were analyzed for the presence of SARS-CoV-2 RNA through the quantification of ORF1b, N1, and N3 gene targets via one-step real-time qPCR. In general, the three RNA targets demonstrated different performances and dynamics over the studied time period, underlying the usefulness of multiple viral targets in the surveillance of SARS-CoV-2 in wastewater. During the first 12 months, the quantification of the selected SARS-CoV-2 viral targets also correlated with the reported clinical cases in the same geographical area; however, from the overall data analysis this did not appear to significantly anticipate the epidemic waves. In conclusion, this study further supports the use of wastewater surveillance as a real time indicator of the human circulation of SARS-CoV-2. Moreover, the use of multiple viral gene targets has been shown to improve the reliability of SARS-CoV-2 surveillance in wastewater over time.

## 1. Introduction

The COVID-19 pandemic has had a profound global impact, prompting the need for complementary approaches to monitor viral dynamics and mitigate the effects of infectious outbreaks. Wastewater-based epidemiology (WBE) has emerged as an effective public health approach, with great potential as an early indicator of pandemic waves due to outbreaks associated with old or emerging infectious pathogens [1]. This population-scale approach can rapidly provide anonymous and aggregated data at a low cost through the passive contribution of the community, regardless of the limitations of clinical testing. As a result, during the COVID-19 pandemic, wastewater surveillance was implemented in many countries worldwide, including Italy [2,3,4].

The ongoing improvements in the standardization and implementation of WBE methodologies, including the selection of appropriate biomarkers, are essential for its effectiveness in public health monitoring. In the case of SARS-CoV-2, international guidelines have recently been published for the detection and quantification of viral RNA from wastewater [5,6]. Although several RNA biomarkers have been proposed, no specific recommendation has been made by the scientific community on the optimal or preferred viral targets to be used for this purpose. The selection of the most appropriate RNA target is crucial not only for the accurate viral load quantification, but also for addressing the challenges posed by viral mutations and emerging variants. Indeed, mutations that affect the primer/probe-binding sites of the SARS-CoV-2 genome can reduce molecular testing performance. Throughout the pandemic, several mutations have been described, resulting in partial or complete PCR target failure [7]. For this reason, the scientific community is presently working on an international standard document (International Organization for Standardization, ISO) defining the general requirements for the reliable detection of SARS-CoV-2 and its variants in wastewater, providing information on different sets of viral-gene targets described in the literature and on possible criteria for their choice. Furthermore, degradation of viral sequences, variations in wastewater and sludge quality, such as the presence of inhibitory substances, and pre-analytical wastewater processing can result in suboptimal conditions influencing the sensitivity and specificity of molecular assays [6]. Unfortunately, not all published reports on SARS-CoV-2 wastewater surveillance provide information on the optimization of pre-analytical and analytical methods and/or quality assurance controls used in viral detection and quantification [8,9,10,11,12]. Most studies have analyzed one or two viral targets, typically N1, N2, and E genes, whilst the use of controls and additional biomarkers has been recommended to reduce the risk of false-negative results and to guarantee the quality of the results [5,6,13,14].

Moreover, the understanding of temporal dynamics and the relationship between different SARS-CoV-2 RNA targets and their association with clinical data is still an active field of research. Assessing correlations among SARS-CoV-2 RNA targets quantified in wastewater and temporal correlations with the reported clinical cases could provide deeper insights into the reliability and the predictive value of WBE in public health surveillance. The present study is part of the “SARI” (“Environmental Surveillance of Wastewater in Italy”) Project, initially launched in July 2020 as a voluntary pilot initiative for monitoring SARS-CoV-2 RNA in wastewater. Following the European Commission’s recommendation, the project was officially established in October 2021, as a nationally funded program, contributing to the enhancement of genomic and epidemiological surveillance. The project, coordinated by the Italian National Institute of Health (ISS), has facilitated the establishment of a network of participating national territorial structures, such as local health authorities, laboratories of universities and research centers, and integrated water service providers, using common standardized validated pre-analytical and analytical methods [15,16].

The present study reports the results of SARS-CoV-2 surveillance from wastewater samples collected weekly from the province of Sondrio (Lombardy region, Italy) over a 2-year period, as part of the national surveillance program (https://www.iss.it/en/cov19-acque-reflue, accessed on 11 October 2024). In particular, the objectives of the study were to evaluate the performance and dynamics of SARS-CoV-2 in wastewater, through the detection and quantification of the ORF1b (nsp14), N1, and N3 gene targets, and to compare the results with the number of weekly reported COVID-19 cases in the same geographical area during the first 12 months of surveillance. The results of this study have shown different dynamics of SARS-CoV-2 gene targets in wastewater during the investigated period supporting the use of multiple viral RNA markers to increase the robustness and the reliability of SARS-CoV-2 wastewater surveillance over time.

## 2. Materials and Methods

A total of 106 wastewater samples were collected weekly, from 2 February 2022 to 7 February 2024, at the Wastewater Treatment Plant (WWTP) of Sondrio (Italy) and processed for the detection and quantification of the ORF1b gene target for the surveillance of SARS-CoV-2 RNA, according to the SARI protocol [15]. A modification of the SARI protocol was used by the laboratories of the Lombardy Region participating in the national wastewater surveillance project, which also included the detection and quantification of two additional gene targets, N1 and N3, following optimization and validation of the pre-analytical and analytical methods [16].

### 2.1. Sample Collection

**Figure 1:**
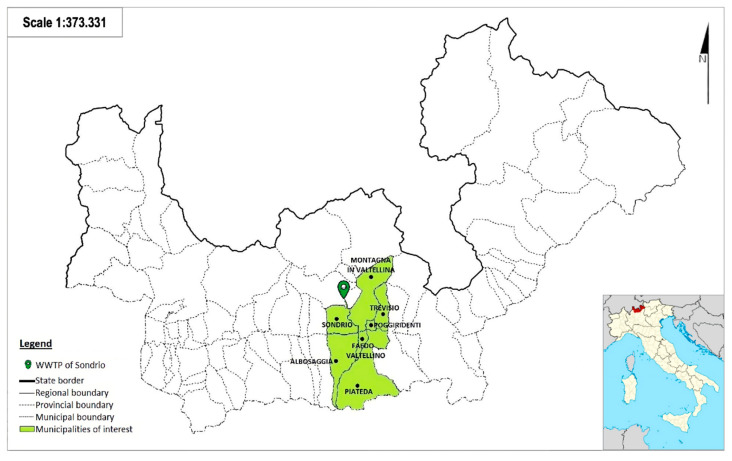
Map of the municipalities (green color) served by the WWTP of Sondrio, Italy. The images were obtained from the provincial geoportal of Sondrio.

The WWTP serves a population of 49,500 equivalent inhabitants, receiving wastewater from seven municipalities (Sondrio, Montagna in Valtellina, Poggiridenti, Tresivio, Faedo Valtellino, Albosaggia, and Piateda), as shown in Figure 1. During the studied period, the WWTP of Sondrio registered an average incoming flow of 13,680 m^3^/s.

By using an auto-sampler, 500 mL of a 24 h-composite sample was collected weekly at the influent of the WWTP during the whole study period. The refrigerated raw sample was transported in polyethylene bottles to the Microbiology Laboratories of the University of Milano-Bicocca for pre-analytical processing and molecular analysis. Wastewater sample analysis was performed by the laboratory generally within 48 h following reception, and results were reported on the national dashboard. Occasionally, samples that could not be analyzed immediately were stored at −20 °C and processed subsequently. The comparison between freshly processed samples and biobanked samples tested within 1 month following collection did not show discordant results in terms of SARS-CoV-2 RNA detection and/or viral load determination.

### 2.2. Viral Concentration

Following heat inactivation at 56 °C for 30 min, the raw sample was concentrated using a polyethylene glycol (PEG)-based method published by Wu and collaborators [17] with minor modifications. Briefly, a 45 mL of sample was spiked with a known amount of Mengovirus (MgV), used as a control of the pre-analytical process, and then centrifuged at 4500× *g* for 30 min at 4 °C. After centrifugation, 40 mL of supernatant was mixed with 4 g of PEG-8000 (Fisher Scientific, Waltham, MA, USA) and 0.9 g of 99.5% NaCl (Sigma-Aldrich, Burlington, VT, USA). The sample was centrifuged at 12,000× *g* for 2 h at 4 °C, and the pellet was resuspended in 750 µL of PBS. The concentrated sample was then further processed for nucleic acid extraction.

### 2.3. Nucleic Acid Extraction

Viral RNA was extracted using the semi-automated NucliSENS^®^ easyMag^®^ system (bioMérieux, Marcy l’Etoile, France). The resuspended pellet underwent lysis for 20 min in 2 mL of NucliSENS Lysis Buffer reagent (bioMérieux, Marcy l’Etoile, France) at room temperature, then 75 µL of magnetic silica beads were added to the sample. The NucliSENS^®^ easyMag^®^ system was set using the “specific B” protocol. Following extraction, nucleic acids were eluted in 100 µL and used for molecular SARS-CoV-2 detection. A second aliquot of extracted material was stored as part of the MicroMiB collection of the University of Milano-Bicocca, an associated partner of the Italian Microbial Resource Research Infrastructure (MIRRI-IT), belonging to the MIRRI-ERIC European Infrastructure, for future analysis.

### 2.4. Viral RNA Quantification

SARS-CoV-2 RNA was detected via one-step real-time quantitative PCR, through the quantification of the ORF1b (nsp14), N1, and N3 gene targets. A list of all primers and probes used in Real-Time qPCR experiments is available as supplementary data [15,18,19] (Table S1). All PCR assays were performed using the TaqMan technology and run on a CFX96 thermocycler (Bio-Rad, Hercules, CA, USA) under the conditions reported in Table S2. In each PCR experiment, 5 µL of sample was used in a 20 µL total mixture. The reaction mixture was composed by 5 µL of UltraPlex 1-Step ThoughMix 4X (Quantabio, Beverly, MA, USA) and a variable volume of Forward Primer, Reverse Primer and DNase-, RNase-, and Protease-free water (Invitrogen, Carlsbad, CA, USA) depending on the studied targets. Cycle threshold (Ct) values were determined using the CFX Maestro Software v2.3 (Bio-Rad, Hercules, CA, USA).

A sample was considered positive when its Ct value was <40. PCR inhibition was used as a quality parameter of the quantification. To verify the presence of PCR inhibitors, Ct values obtained from the sample with the addition of 1 µL of a 1.0 × 10^3^ gene copies/µL of CoV-2 RNA nsp14 were compared with the Ct value of water (Invitrogen, Carlsbad, CA, USA) containing the same 1 µL volume of CoV-2 RNA nsp14, according to the following formula: ∆Ct = Ct (sample + spike RNA) − Ct (water + spike RNA). The sample was considered acceptable if ∆Ct was ≤2. Viral RNA quantification was calculated by the use of standard curves determined using 10-fold dilutions of both the synthetic CoV-2 dsDNA construct carrying SARS-CoV-2 sequences including ORF1b sequence, gently supplied by the Italian National Institute of Health (ISS), and of synthetic RNA fragments containing N1 and N3 sequences (Material ID: 10169), produced and distributed by the National Institute of Standards and Technology (NIST) [20]. Standard curves were used to convert Ct values detected in wastewater samples into SARS-CoV-2 RNA gene copies/µL per reaction. Only standard curve slope values ranging from −3.1 to −3.6 were considered acceptable with an R^2^ value ≥ 0.98. Finally, viral load was expressed as gene copies per liter (GC/L).

### 2.5. Data Normalization

The GC/L values were normalized according to daily incoming wastewater flow and the population served by the WWTP and then expressed as GC/(day ∗ inhabitant equivalent). Data on the incoming flow was provided by the personnel of the WWTP. Flow data were not always available for every sample collected. To address this limitation, an estimation method devised by Biomathematics and Statistics Scotland (BioSS) was employed. This approach estimated flow for the normalization process based on the historical average flow and predicted ammonia levels. Detailed descriptions of the modeling methods calculated with R software (R Core Team 2021 v. 4.4.0) can be found in the normalization protocol available on protocols.io [21].

### 2.6. Viral Sequencing

Nucleic acids were sent monthly (flash survey) to the ISS reference laboratory for sequencing, as required by the SARI project. Flash surveys are rapid tests carried out once a month, usually in the first week. Specifically, from February 2022 to January 2024, 22 samples from the WWTP of Sondrio were sent to the ISS for sequencing purposes. Rapid screening for variants was performed by an RT-PCR followed by amplicon sequencing, as previously described [22].

### 2.7. Statistical Analysis

All data were initially log-transformed, plotted for the removal of outliers and then fitted with a regression model using R to assess general trends of the targets measured. The Agresti–Coull interval was used to estimate 95% confidence intervals (CIs) calculated with the Binom package [23]. Statistical significance was defined as a *p*-value less than 0.05. Student’s *t* test was used for mean comparisons, whereas the Chi-squared test with the Yates correction and Fisher’s exact test were performed for frequency analysis. Correlations were established using the Spearman ρ correlation, and Fisher’s r-to-z transformation was used to compare correlation coefficients.

## 3. Results

Overall, a total of 106 weekly collected wastewater samples from the WWTP of Sondrio were analyzed for the presence of SARS-CoV-2 RNA as well for viral quantification through the use of ORF1b (nsp14), N1, and N3 gene targets.

**Figure 2:**
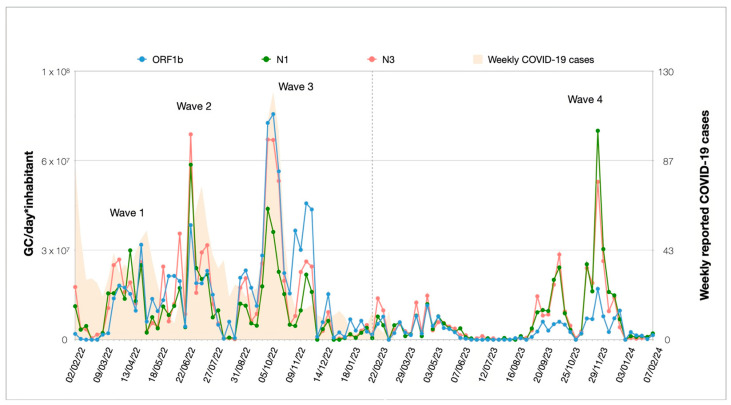
Weekly trends in the loads of ORF1b, N1, and N3 from the WWTP of Sondrio (Italy) during the 2-year surveillance and COVID-19 cases reported within the municipalities. The dotted grey line delimits the 2022–2023 and the 2023–2024 intervals.

As shown in Figure 2, the highest viral loads were reported on June 2022 for N3 (7.26 × 10^7^ GC/day ∗ inhabitant), October 2022 for ORF1b (7.97 × 10^7^ GC/day ∗ inhabitant), and November 2023 for N1 (7.38 × 10^7^ GC/day ∗ inhabitant). Four distinct waves are notable over the 2-year wastewater surveillance period. Three of them (wave 1, 2, and 3) align with peaks registered by COVID-19 clinical surveillance, from data available only for the first 12-month time period.

**Figure 3:**
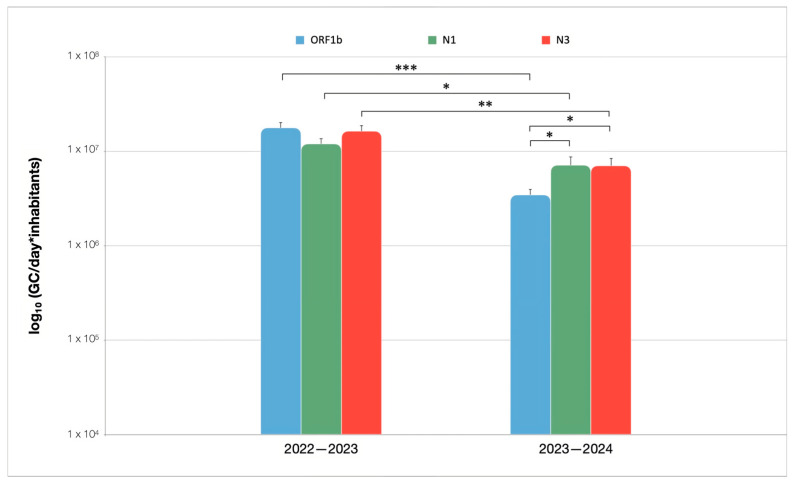
Average load of ORF1b, N1 and N3 in wastewater samples collected at the WWTP of Sondrio (Italy). Results are divided into 2022–2023 (*n* = 53) and 2023–2024 (*n* = 53) intervals and expressed with logarithmic scale as mean ± standard error; * *p* < 0.05; ** *p* < 0.01; *** *p* < 0.001.

Average loads of SARS-CoV-2 RNA targets are presented in Figure 3. In the first monitoring period, no significant differences were observed in viral quantification using the three different gene targets. During the first 12 months of wastewater surveillance, the ORF1b gene target resulted in the highest loads (1.77 × 10^7^ GC/day ∗ inhabitant), but its levels decreased significantly (*p* < 0.001) during the subsequent period. Notably, during this second time period, the average viral loads of ORF1b (3.46 × 10^6^ GC/day ∗ inhabitant) were significantly lower than those detected using N1 (7.14 × 10^6^ GC/day ∗ inhabitant) and N3 (7.04 × 10^6^ GC/day ∗ inhabitant) (*p* < 0.05 for both N1 and N3). However, in general, a significant reduction in the quantification of all three gene targets was observed in the second monitoring period, as compared to the first monitoring period (*p* < 0.05 for N1; *p* < 0.01 for N3). The *p*-values are summarized in Table S3.

In the first monitoring period, all SARS-CoV-2 RNA targets quantitatively determined comparable viral loads. During the second surveillance period, viral quantification in wastewater through the use of the N1 and N3 gene targets showed almost identical viral load levels, which were found to be higher than those detected through the use of ORF1b (Figure 3). Differences in viral load determination through the quantification of ORF1b, N1, and N3 during the study period can be attributed to several variables such as epidemiological, methodological, or environmental factors. Ongoing investigations are intended to clarify this phenomenon.

### 3.1. Frequency Analysis

The frequency analysis of positive wastewater samples was conducted to assess the contribution of viral RNA targets in qualitative detection of SARS-CoV-2 in wastewater. During the 2-year surveillance, 102 out 106 (96%) of wastewater samples were positive for at least one viral RNA target.

**Figure 4:**
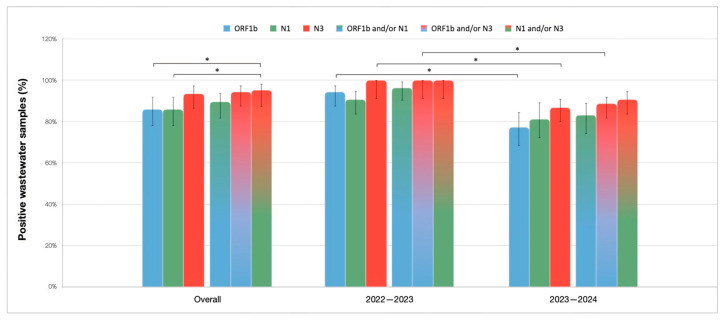
Percentage of positive wastewater samples for ORF1b, N1, N3 and their combinations in the overall analyzed period (*n* = 106) and divided in the 2022–2023 (*n* = 53) and 2023–2024 (*n* = 53) intervals. Results are expressed as percentage ± 95% Agresti-Coull interval; * *p* < 0.05.

Overall, the detection of the N3 gene was observed in the highest number of wastewater samples (99/106, 93%); however, no significant difference was found in the positivity rates of this target compared to ORF1b and N1 (both 91/106, 86%), as shown in Figure 4. When gene targets were combined, the N1 and/or N3 pair reached a 95% (101/106) detection rate, followed by the ORF1b and/or N3 (100/106, 94%) and the ORF1b and/or N1 (95/106, 90%) combinations. A statistically significant increase (*p* < 0.05) in the detection was observed using the N1 and/or N3 target combination compared to N1 and ORF1b alone. A detailed list of *p*-values is available (Table S4).

In the first monitoring period, the positivity of wastewater samples for at least one target was 100% (53/53), whereas the positivity rate was reduced (49/53, 92%) during the 2023–2024 period. In 2022–2023, all wastewater samples (53/53, 100%) were positive for N3. However, a significant reduction (*p* < 0.05) in the positivity rate for this target (46/53, 87%) was observed during the subsequent time period. Notably, there was a general reduction in positivity rates for all three targets during 2023–2024 as compared to the previous period, although the greatest reduction was observed for ORF1b, whose detection rate decreased significantly (*p* < 0.05) from 94% (50/53) to 77% (41/53). The N1 gene target did not show such a marked reduction between the two time periods.

The ORF1b and/or N3 and N1 and/or N3 combinations exhibited the highest detection rates in both analyzed time periods. The ORF1b and/or N1 combination was more affected by the reduction in positivity during 2023–2024. However, the ORF1b and/or N3 combination decreased significantly (*p* < 0.05) in 2023–2024, further supporting the significant reduction in the individual frequency for both ORF1b and N3. However, no statistical significance was observed within each of the two investigated time periods, when analyzing the three gene targets alone or in combination, supporting the ability of the studied gene targets to reliably qualitatively mark the presence of SARS-CoV-2 in wastewater (Figure 4).

### 3.2. Correlation Analysis Between SARS-CoV-2 RNA Targets in Wastewater

**Figure 5:**
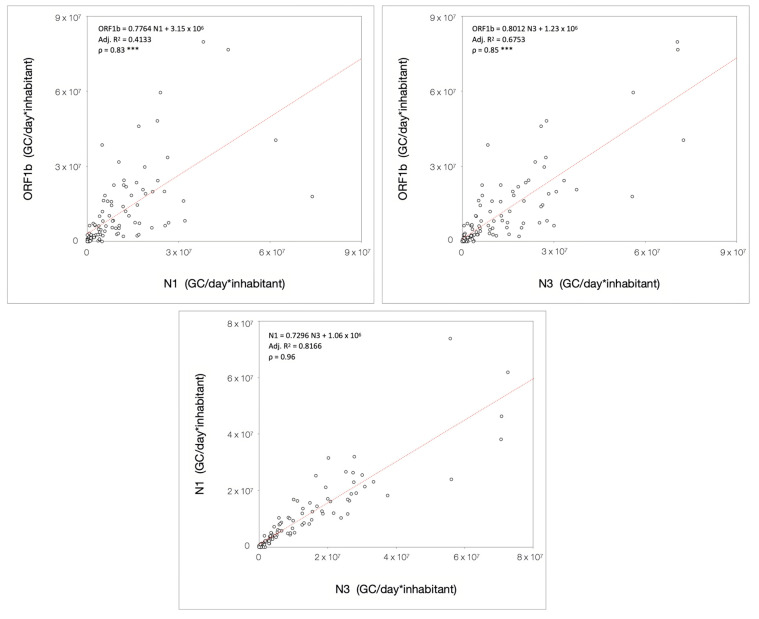
Scatter plot of the loads for all combinations of analyzed gene targets, fitted with a regression model and tested for Spearman’s correlation. The plots represent all three pair-wise relationships in the overall studied period; *** *p* < 0.001 vs. N1/N3.

The Spearman correlation analysis revealed that the load of SARS-CoV-2 RNA in wastewater was significantly correlated (*p* < 0.001) among gene targets. As shown in Figure 5, similar overall correlations were found for ORF1b and N1 (ρ = 0.83) and for ORF1b and N3 (ρ = 0.85), which were found to be significantly reduced (*p* < 0.001 for both ORF1b/N1 and ORF1b/N3) as compared to the N1 and N3 correlation (ρ = 0.96). The R^2^ values indicated a linear relationship between N1 and N3, where an increase (or a decline) in one target was associated with a rise (or decline) in the other. By contrast, the overall relationship between ORF1b and the two N genes was not strictly linear, especially for ORF1b and N1. Indeed, about 60% of the variability of ORF1b load in wastewater cannot be explained by the model, when compared to N1. These results imply that diverse factors play a key role in ORF1b dynamics.

**Figure 6:**
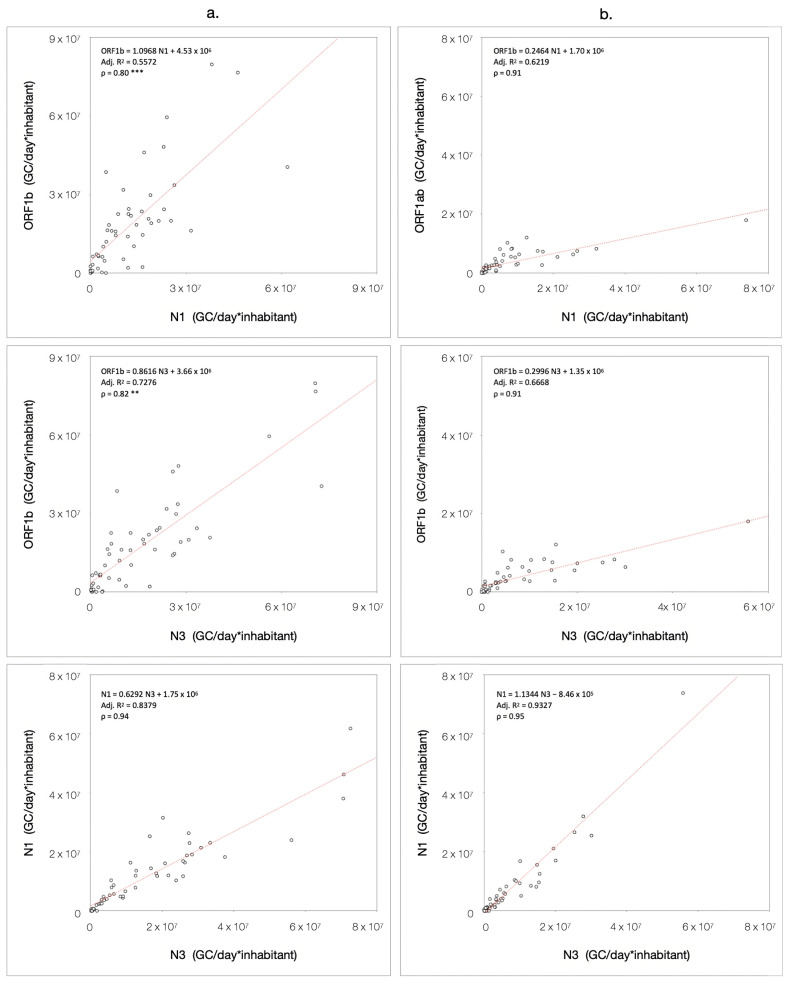
Scatter plot of normalized load for all combinations of RNA gene targets, fitted with the regression model and tested for Spearman’s correlation. The plots represent all three pair-wise relationship and are divided in 2022–2023 (**a**) and 2023–2024 (**b**) intervals; ** *p* < 0.01 vs. N1/N3 in the same period, *** *p* < 0.001 vs. N1/N3 in the same period.

The correlations observed in the 2022–2023 and 2023–2024 periods are presented in Figure 6. In the first monitoring period (Figure 6a), the correlation between ORF1b and N1 (ρ = 0.80) and ORF1b and N3 (ρ = 0.82) was significantly lower than that of N1 and N3 (ρ = 0.94) (*p* < 0.001 for ORF1b/N1; *p* < 0.01 for ORF1b/N3). These differences can be attributed to the continuous inversions of trends that characterize the three different gene targets during the first 12-month surveillance (Figure 2). By contrast, differences in the correlation between the gene targets were detected in the second monitoring period (Figure 6b), with similar correlation coefficients for all target pairs (ρ = 0.91 for ORF1b/N1 and ORF1b/N3; ρ = 0.95 for N1/N3), indicating that their relationship varied considerably across the entire period of surveillance. During the second period, a reduction in significant differences suggested that the relationship of SARS-CoV-2 targets in wastewater changed until a certain stability among relative Spearman’s ranks had been achieved. This stability resulted in an increase in correlation coefficients compared to the previous period. Nevertheless, during 2023–2024, a significant increase (*p* < 0.05) in the correlation between ORF1b and N1 was documented, indicating a noticeable shift in their dynamics. Statistical significance resulting from the correlation analysis and the coefficients comparison is listed in Tables S5–S7.

### 3.3. Correlation Analysis Between SARS-CoV-2 RNA Targets and Reported COVID-19 Cases

Data on notified COVID-19 cases during the first 13 months of the wastewater surveillance period was retrieved from the Lombardy regional database. Testing rates changed during the study period, with a high number of tests being performed at the beginning of 2022 and decreasing thereafter (Figure S1), resulting from the reduction in COVID-19 cases (Figure 2).

**Figure 7:**
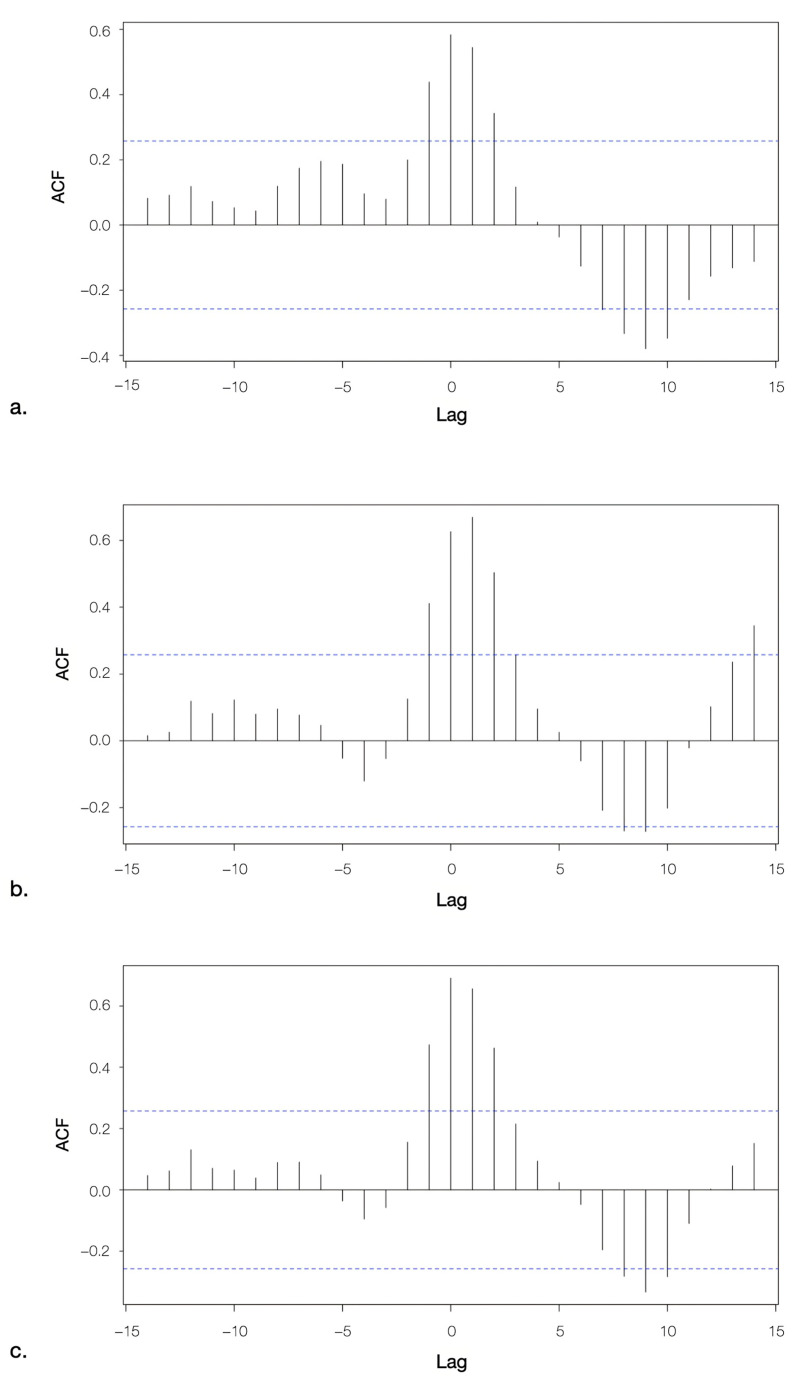
Cross-correlogram between ORF1b (**a**), N1 (**b**), and N3 (**c**) and weekly reported COVID-19 cases within the catchment area of the WWTP of Sondrio (Italy). Each lag time corresponds to 7 days. The horizontal dashed lines indicate the 95% confidence thresholds at ±0.26.

The application of a cross-correlation model enabled the estimation of temporally significant relationships between the quantification of SARS-CoV-2 RNA in wastewater and the reporting of COVID-19 cases by clinical surveillance within the catchment area of the WWTP. The lags depicted in Figure 7 correspond to weekly intervals, and the dotted lines indicate the thresholds of statistical significance (±0.26). A value of cross-correlation function (CCF) exceeding the threshold of significance suggests a statistically significant lag. In general, qPCR amplification signals for all viral targets detectable in wastewater temporally correlated with the weekly reported COVID-19 clinical cases within the municipalities under investigation. Indeed, a strong positive correlation without any temporal delay (lag 0) was found to be significant for the three viral targets. A significant positive correlation was observed for N1 at lag 14 (Figure 7b). Furthermore, a significant negative correlation was observed for all targets at approximately lag 9; these moderate correlations would require further investigation.

**Figure 8:**
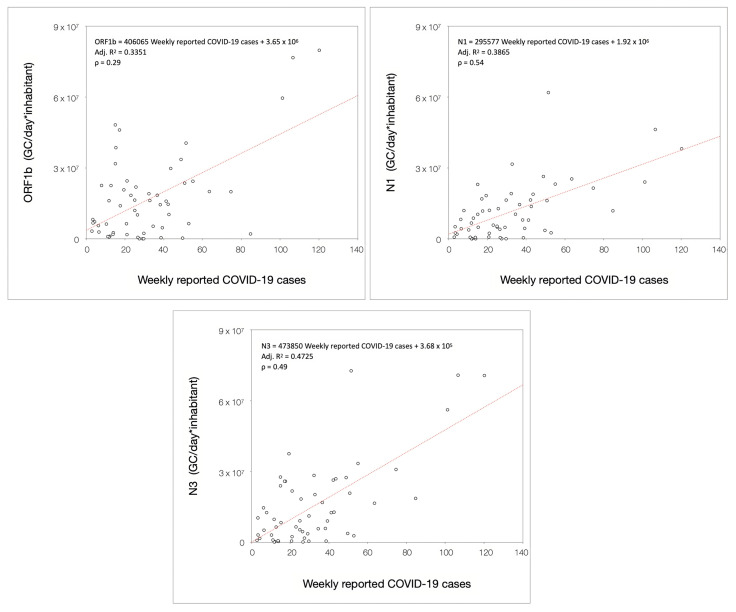
Scatter plot of normalized load for all combinations of viral RNA gene targets and weekly COVID-19 cases reported by clinical surveillance. Data were fitted with the regression model and tested for Spearman’s correlation. The plots represent all the three pair-wise relationships with reported cases.

The Spearman correlation analysis confirmed that the quantification of SARS-CoV-2 RNA in wastewater was significantly correlated (*p* < 0.05 for ORF1b; *p* < 0.001 for N1 and N3) with the number of reported COVID-19 clinical cases. Results indicated varying degrees of correlation across viral targets. Among these, ORF1b exhibited a very low correlation with data on clinical cases (ρ = 0.29), as illustrated in Figure 8. Results indicate that the 70% variability in the load observed through the use of ORF1b cannot be explained by the model. In contrast, the quantification of N1 in wastewater displayed a modest correlation (ρ = 0.54), indicating that, among the viral gene targets investigated, this represents the one that mostly correlated with the reported number of COVID-19 cases. Similarly, N3 demonstrated a mild correlation with the number of reported cases (ρ = 0.49), even if the model can explain about half of the variability of N3 in wastewater. It must also be acknowledged that the variability not accounted for in the model includes asymptomatic individuals or those who were not diagnosed and/or reported with COVID-19 infection, indicating other potential factors influencing epidemiological surveillance. This underlines that the model cannot fully explain 100% of the variability in the observed viral loads measured in wastewater. However, no statistically significant differences were observed in the correlation between viral targets and the number of reported cases. Refer to Tables S8 and S9 for the *p*-values from the correlation analysis and the comparison of correlation coefficients.

During the analysed period, the reporting delay for documented COVID-19 cases remained consistently low, with an average of approximately 2 days (Figure S2). This consistent, minimal delay was maintained throughout the analyzed time frame, ensuring that any potential impact on the correlation analyses would be negligible, thereby supporting the robustness and reliability of the correlation findings.

### 3.4. Viral Sequencing Analysis

A total of 22 samples were sent during the 24-month surveillance period, on a monthly basis, to the ISS for sequencing purposes. Unfortunately, only 3 samples collected in February, July and September 2022 were successfully sequenced and variants attributed to Omicron BA1.2 and BA4/BA5. Sequencing was unsuccessful for the remaining samples, probably due to low viral loads. However, during the same surveillance period a large number of variants were identified in wastewater samples by ISS from various wastewater treatment plants throughout the Lombardy region, as part of the national wastewater surveillance project. These variants included, in addition to those identified in Sondrio: BQ.1, BN.1, XBB.1.5*, CH.1.1, XBB.2.3*, and CM.7. It is important to note that the target region for Sanger sequencing is a long fragment of about 1500 nucleotides of the spike protein, which is a different target from that used for virus quantification.

## 4. Discussion

Wastewater-based epidemiology has been shown to provide a cost-effective, non-invasive, and population-wide approach to assess the circulation and potential epidemic waves associated with infectious agents [24]. This approach offers several advantages over traditional clinical testing, as it captures infection trends regardless of individuals’ testing behavior and includes undiagnosed or asymptomatic cases. The application of WBE for the surveillance of SARS-CoV-2 has proven particularly valuable during the emergence of COVID-19 pandemic [1,2,3,4,25,26], particularly when clinical testing fluctuated or diminished in time, also providing data on the emergence and circulation of new viral variants. Despite its benefits, several challenges may influence the use of wastewater surveillance as an indicator of epidemic waves in the population. Environmental factors, such as rainfall and increased temperatures, have been shown to significantly impact viral detection sensitivity, by lowering viral RNA levels, and/or reducing viral stability in wastewater, resulting in the degradation of SARS-CoV-2 RNA [27,28]. Variable physical-chemical properties of wastewater, such as pH, ammonia, total solids, presence of PCR inhibitors, population size of the catchment areas, and different proportions of flow from urban, agriculture, and industrial sources, can also influence the use of wastewater surveillance as an epidemiological tool [29]. Moreover, the selection of RNA viruses’ gene targets can impact on the long-term monitoring and accurate viral load determination in wastewater, where the high rates of genetic mutations and the emergence of viral variants can reduce the efficiency of viral fragments’ amplification [6,30,31,32]. For example, the complete loss of detection of the spike gene target used by the diagnostic Thermo Fisher Scientific TaqPath COVID-19 assay (Thermo Fisher Scientific, Waltham, MA, USA) has resulted in reporting false-negative results, although it represented a hallmark of the Alpha (B.1.1.7) variant allowing for sequence-free estimation of Alpha’s emergence and prevalence in early 2021 [31,32]. However, such mutations can reduce sensitivity or determine false-negative results for specific viral targets in wastewater. In this context, a careful selection of RNA viral markers is critical to ensure reliable detection and quantification. Moreover, the use of multiple RNA markers targeting conserved regions of the viral genome has been recommended [5,6,14]. Ongoing studies on SARS-CoV-2 wastewater dynamics are also focusing on the ability of viral loads to correlate with clinical epidemiological surveillance [33,34,35,36]. This highlights the ongoing need, as viruses evolve, for updating WBE detection protocols in order to provide robust data to detect outbreaks and track variants for the implementation of public health measures and containment of epidemic and pandemic events [37].

In this study, wastewater surveillance was performed for a 2-year monitoring period using standardized pre-analytical and analytical protocols for the detection and quantification of three different viral RNA gene targets—ORF1b, N1, and N3. To mitigate the potential degradation of the viral genome, almost all samples tested in this study were processed within 48 h or stored immediately at −20 °C and analyzed within 1 month. In addition, three different viral gene targets were used in parallel to evaluate the frequency and dynamics of SARS-CoV-2 detection in wastewater, where multiple viral variants may be concomitantly present in wastewater samples, harboring alterations in specific genomic regions that can affect the performance of primers and probes used for viral quantification. The high correlation observed between viral RNA targets suggests that all targets consistently reflect the presence of SARS-CoV-2 in wastewater. Furthermore, the frequency and quantification analysis of the three viral RNA targets was used to determine the best combination of gene targets, with the aim to maximizing WBE’s effectiveness in delivering accurate results. Positivity rates for ORF1b, N1, and N3, showed no significant difference within the same time period, suggesting that all three targets are equally useful in the qualitative determination of SARS-CoV-2 in wastewater. However, results indicated that the combination of N1 and/or N3 accounted for higher positivity rates compared to N1 and ORF1b alone. This finding further supports the simultaneous use of target combinations to improve detection rates.

Overall, the correlation analysis used to assess the relationships between viral loads of the three investigated viral gene targets showed different dynamics during the 2-year period of wastewater monitoring. Initially, the relationship between N1 and N3 was found to be relatively stable, whilst ORF1b/N3 and ORF1b/N1 showed a different behavior. However, in the second period, the correlation between ORF1b and N1 varied, as well as that between ORF1b and N3, supporting the possibility that the dynamics between ORF1b and the N1/N3 gene targets shifted during the surveillance period. This suggests that the quantification of ORF1b may have been influenced by the introduction/emergence of new variables within the circulating viral genomes. This divergence could be associated with a reduced amplification efficiency due to changes in the nucleotide sequence of new circulating viral variants, as reported in several previous studies [38,39,40,41,42], which have also included the ORF1 gene target [31,32,43]. Other potential causes include the overexpression of the N genes that could result from selective advantages of certain variants leading to higher transcription levels of these targets [44,45]. As wastewater is a composite sample containing a pool of viral genomes originating from infected individuals in the WWTP catchment area, detection and viral load determination needs to take into account a broader genomic diversity compared to clinical samples. This heterogeneity may amplify the variability in detection efficiency for different gene targets, particularly in the presence of co-circulating variants with distinct genetic signatures. Unfortunately, sequencing analysis was only successful in a limited number of samples and did not include the viral gene targets used for viral load determination. Further studies are therefore needed to validate these assumptions. Moreover, the influence of environmental and physico-chemical factors makes wastewater a more complex matrix compared to biological samples, with multiple factors potentially accounting for greater uncertainty or variability in the measured signals [46]. Managing this uncertainty requires a multidisciplinary approach to ensure the successful implementation of WBE as a public health tool.

The role of wastewater-based epidemiology as a complementary surveillance tool in providing community-level insights into the prevalence of viral infections is based on the capacity to correlate viral loads in wastewater with the number of clinical cases, as a predictive model of viral transmission. Clinical data were collected for the first 13 months of the study, during which testing rates varied from the beginning of 2022, when the circulation of SARS-CoV-2 in the population was high and decreased thereafter with the end of the COVID-19 emergency state being declared in Italy on the 31st of December 2022. The testing and reporting of clinical cases fell in subsequent months, making the correlation with wastewater surveillance data unreliable and therefore not included in the second part of the monitored period. During the first part of the study, the reporting delay between symptoms’ onset and testing for the diagnosed SARS-CoV-2 infections remained consistently low (approximately 2 days). This stability in the reporting delay over the analyzed time frame ensures that it is unlikely to have introduced significant bias in the correlation analyses. By maintaining a constant and minimal delay, the reliability of the temporal alignment between wastewater data and infection case reports is preserved, reinforcing the validity of the observed correlations. As reported in several previous studies [9,33,47,48,49], the surveillance of SARS-CoV-2 in wastewater has been shown to allow the prediction of infection dynamics within the population in the WWTP catchment area, supporting the use of wastewater as an early detection tool of epidemic waves. In the present study, the cross-correlation analysis has confirmed a strong, positive correlation between reported cases and the viral load of SARS-CoV-2 in wastewater without anticipating the epidemic waves of infection, when clinical data were available. Nonetheless, the quantification of N1 and N3 in wastewater reflected the highest correlation with the reported COVID-19 cases.

The extended 24-month period and the standardized pre-analytical and analytical approach in wastewater processing have provided robust data to evaluate target performance and dynamics. Nevertheless, this study has some limitations. Environmental factors, such as rainfall and outdoor temperature, which could have had an impact on the sensitivity of SARS-CoV-2 detection were not taken into account by the present wastewater surveillance protocol. Chemical indicators in wastewater, such as total ammonia and phosphate, have been reported to calibrate/level out the dilution effect of rainfalls, and their potential use in the future would allow to improve wastewater surveillance capacity. Moreover, in this study SARS-CoV-2 viral load was normalized based on the population size in the WWTP catchment area. The future use of the Pepper mild mottle virus (PMMoV) as a fecal indicator, or alternative methods for the estimation of the population size, may provide a better evaluation of the dynamics of different viral gene targets and improve their correlation with the reported number of clinical cases. In this context, the analyses of wastewater from high-mobility hubs, such as bus stations and trains, could have provided additional useful information in tracking the local viral circulation. Another important drawback of the study is the limited clinical data, available only for the first part of the surveillance period, to assess the correlation between viral loads in wastewater and COVID-19 cases, as well as the variability in the testing rates during the first part of the monitoring period. Finally, the analysis of circulating variants in the area of Sondrio was conducted only on a monthly basis, providing identification of SARS-CoV-2 variants in only three samples during the whole monitoring period. The lack of information on the locally circulating viral variants and on the potential mutations within the viral gene targets occurring during the study period has not allowed us to better understand or explain the divergence in the dynamics of ORF1b detection observed during the second monitoring period.

## 5. Conclusions

In conclusion, this study highlights the importance of using multiple viral targets to ensure robust and reliable detection and quantification of SARS-CoV-2 in wastewater surveillance. This approach not only supports its application as a real time indicator for tracking the spread of SARS-CoV-2 in populations but also demonstrates the stability and reliability of the findings despite inherent uncertainties in wastewater data.

## Data Availability

The original contributions presented in this study are included in the article/Supplementary Material. Further inquiries can be directed to the corresponding author.

## Supplementary Materials

The following supporting information can be downloaded at: https://www.mdpi.com/article/10.3390/microorganisms13020236/s1, Table S1: Specifications on primers and probes used in this study; Table S2: Thermal profiles used in Real-Time qPCR experiments; Table S3: *p*-values resulted from the comparison of the average loads of SARS-CoV-2 gene targets expressed as GC/day ∗ inhabitants; Table S4: *p*-values obtained from the frequency analysis of SARS-CoV-2 gene targets and their combinations; Table S5: *p*-values resulted from the correlation analysis between SARS-CoV-2 gene targets quantified in wastewater; Table S6: Values of Z and *p*-values obtained from the Fisher’s r-to-z transformation in the comparison of correlation coefficients resulted from the analysis between the loads of SARS-CoV-2 gene target quantified in wastewater; Table S7: Values of Z and *p*-values obtained from the Fisher’s r-to-z transformation in the comparison of correlation coefficients resulted from the analysis between the loads of SARS-CoV-2 gene targets quantified in wastewater in the two period analyzed; Table S8: *p*-values resulted from the correlation analysis between SARS-CoV_2 gene targets quantified in wastewater and reported COVID-19 cases; Table S9: Values of Z and *p*-values obtained from the Fisher’s r-to-z transformation in the comparison of correlation coefficients resulted from the analysis of SARS-CoV-2 gene targets and reported COVID-19 cases. Figure S1: Testing rates during the analyzed period; Figure S2: Average reporting delay between symptoms onset and testing.

## Abbreviations

The following abbreviations are used in this manuscript:

WBE	Wastewater-based epidemiology
SARI	Sorveglianza Ambientale Reflui in Italia—Environmental Surveillance of Wastewater in Italy
ISS	Istituto Superiore di Sanità- Italian National Institutes of Health
