# Supplementary material for: Wastewater Surveillance for SARS-CoV-2 in Northern Italy: An Evaluation of Three Different Gene Targets
Source: Microorganisms. 2025 Jan 22;13(2):236. doi: 10.3390/microorganisms13020236 (PMC11857900; doi:10.3390/microorganisms13020236)

Supplementary data

Table S1. Specifications on primers and probes used in this study.

LABEL NAME	SEQUENCE	FINAL CONCENTRATION	REFERENCES
2297 (COV-2-F)	ACATGGCTTTGAGTTGACATCT	500 nM	[18]
2298 (COV-2-R)	AGCAGTGGAAAAGCATGTGG	900 nM	[18]
2299 (COV-2-P)	FAM-CATAGACAACAGGTGCGCTC-BHQ1	250 nM	[18]
2019-nCoV_N1-F	GACCCCAAAATCAGCGAAAT	500 nM	[19]
2019-nCoV_N1-R	TCTGGTTACTGCCAGTTGAATCTG	500 nM	[19]
2019-nCoV_N1-P	FAM-ACCCCGCATTACGTTTGGTGGACC-BHQ1	125 nM	[19]
2019-nCoV_N3-F	GGGAGCCTTGAATACACCAAAA	500 nM	[19]
2019-nCoV_N3-R	TGTAGCACGATTGCAGCATTG	500 nM	[19]
2019-nCoV_N3-P	FAM-AYCACATTGGCACCCGCAATCCTG-BHQ1	125 nM	[19]
Mengo 110 (FW)	GCGGGTCCTGCCGAAAGT	500 nM	[15]
Mengo 11 (REV)	GAAGTAACATATAGACAGACGCACAC	900 nM	[15]
Mengo 147 (P)	FAM-ATCACATTACTGGCCGAAGC-BHQ1	250 nM	[15]

Table S2. Thermal profiles used in Real-Time qPCR experiments.

NUMBER OF CYCLES	TEMPERATURE	DURATION
1	50°	30 min
1	95°	3 min
45	95°	10 sec
45	60° for ORF1b and MgV;<br>55° for N1 and N3	45 sec<br>30 sec

Table S3. P-values resulted from the comparison of the average loads expressed as GC/day*inhabitants.

	ORF1b	N1	N3	ORF1b vs N1	ORF1b vs N3	N1 vs N3
PERIOD	p-value	p-value	p-value	p-value	p-value	p-value
Overall	-	-	-	0.595	0.603	0.277
22-23	<0.001	0.043	0.002	0.065	0.694	0.160

23-24				0.037	0.019	0.963

Table S4. P-values obtained from the frequency analysis of SARS-CoV-2 gene targets and their combinations.

PERIOD	TEST	TARGETS AND THEIR COMBINATIONS	ORF1b	N1	ORF1b and/or N1	ORF1b and/or N3
			p-value	p-value	p-value	p-value
Overall (n = 106)	Chi-squared with Yates' continuity correction	**ORF1b**	1	1	0.530	0.066
		**N1**	1	1	0.530	0.066
		**N3**	0.115	0.115	0.460	1
		**ORF1b and/or N1**	0.530	0.530	1	0.312
		**ORF1b and/or N3**	0.066	0.066	0.312	1
		**N1 and/or N3**	0.035	0.035	0.194	1
22-23 (n = 53)	Fisher's exact test	**ORF1b**	1	0.716	1	0.243
		**N1**	0.716	1	0.437	0.057
		**N3**	0.243	0.057	0.495	1
		**ORF1b and/or N1**	1	0.437	1	0.495
		**ORF1b and/or N3**	0.243	0.057	0.495	1
		**N1 and/or N3**	0.243	0.057	0.495	1
23-24 (n = 53)	Chi-squared with Yates' continuity correction	**ORF1b**	1	0.811	0.626	0.196
		**N1**	0.811	1	1	0.416
		**N3**	0.311	0.597	0.786	1
		**ORF1b and/or N1**	0.626	1	1	0.577
		**ORF1b and/or N3**	0.196	0.416	0.577	1
		**N1 and/or N3**	0.112	0.265	0.389	1

Table S5. P-values resulted from the correlation analysis between SARS-CoV-2 gene targets quantified in wastewater.

	**ORF1b/N1**	**ORF1b/N3**	**N1/N3**
**PERIOD**	**p-value**	**p-value**	**p-value**
Overall	<0.001	<0.001	<0.001
22-23	<0.001	<0.001	<0.001
23-24	<0.001	<0.001	<0.001

Table S6. Values of Z and p-values obtained from the Fisher's r-to-z transformation in the comparison of correlation coefficients resulted from the analysis between the loads of SARS-CoV-2 gene targets quantified in wastewater.

		**ORF1b/N1**		**N1/N3**	
**PERIOD**	**TARGET**	**Z**	***p*-value**	**Z**	***p*-value**
Overall	**ORF1b/N1**	0	1	-5.464	<0.001
	**ORF1b/N3**	0.505	0.613	-4.959	<0.001
2022-2023	**ORF1b/N1**	0	1	-3.376	<0.001
	**ORF1b/N3**	-0.318	0.750	-3.058	0.002
2023-2024	**ORF1b/N1**	0	1	-1.613	0.107.
	**ORF1b/N3**	0.052	0.958	1.665	0.096

Table S7. Values of Z and p-values obtained from the Fisher's r-to-z transformation in the comparison of correlation coefficients resulted from the analysis between the loads of SARS-CoV-2 gene targets quantified in wastewater in the two periods analyzed.

	**ORF1ab/N1**		**ORF1ab/N3**		**N1/N3**	
**PERIOD**	Z	p-value	Z	p-value	Z	p-value
2022-2023 vs 2023-2024	-2.045	0.041	-1.675	0.094	-0.282	0.778

Table S8. P-values resulted from the correlation analysis between SARS-CoV-2 gene targets quantified in wastewater and weekly reported COVID-19 cases.

**ORF1b/Weekly reported COVID-19 cases**	**N1/Weekly reported COVID-19 cases**	**N3/Weekly reported COVID-19 cases**
**p-value**	**p-value**	**p-value**

0.028	<0.001	<0.001

Table S9. Values of Z and p-values obtained from the Fisher's r-to-z transformation in the comparison of correlation coefficients resulted from the analysis of SARS-CoV-2 gene targets and weekly reported COVID-19 cases.

	**ORF1b/Weekly reported COVID-19 cases**		**N3/Weekly reported COVID-19 cases**	
	**Z**	***p*-value**	**Z**	***p*-value**
**ORF1b/Weekly reported COVID-19 cases**	0	1	-1.282	0.200
**N1/Weekly reported COVID-19 cases**	-1.632	0.103	0.350	0.726

Figure S1. Testing rates during the analyzed period.

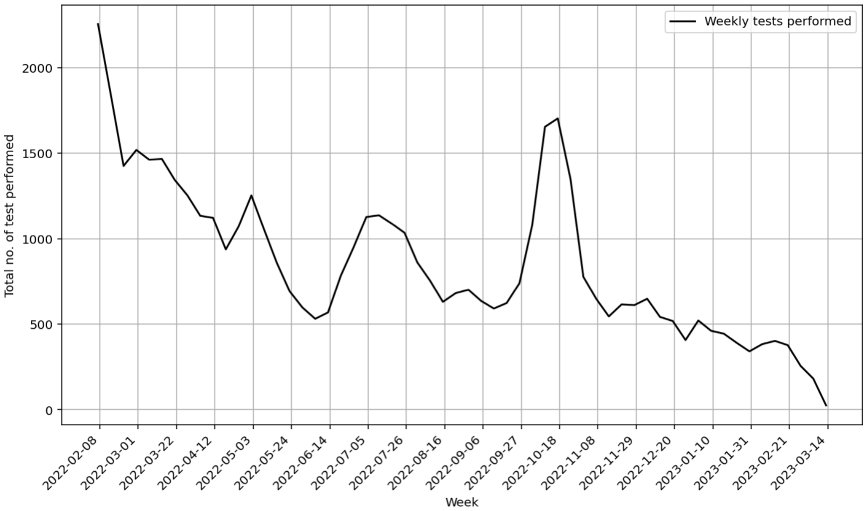

Figure S2. Average reporting delay between symptoms onset and testing.

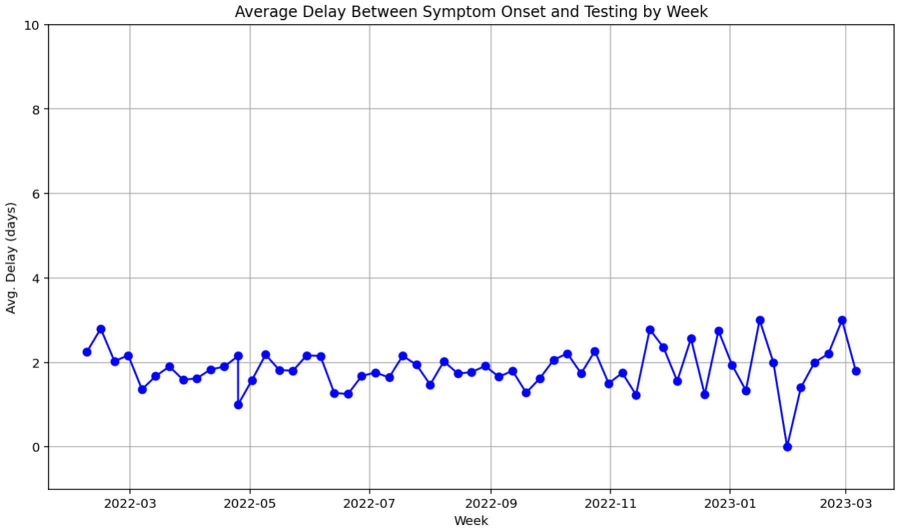

Supplement: Supplementary file 1 [file microorganisms-13-00236-s001.zip › microorganisms-3431647-supplementary.pdf]
